# Two years later – Revisiting autobiographical memory representations in vmPFC and hippocampus

**DOI:** 10.1016/j.neuropsychologia.2017.05.014

**Published:** 2018-02

**Authors:** Heidi M. Bonnici, Eleanor A. Maguire

**Affiliations:** a School of Psychology, University of East Anglia, Norwich NR4 7TJ, UK; b Wellcome Trust Centre for Neuroimaging, Institute of Neurology, University College London, 12 Queen Square, London WC1N 3BG, UK

**Keywords:** vmPFC, Hippocampus, Autobiographical memory, Consolidation, MVPA, Longitudinal

## Abstract

A long-standing question in memory neuroscience concerns how and where autobiographical memories of personal experiences are represented in the brain. In a previous high resolution multivoxel pattern analysis fMRI study, we examined two week old (recent) and ten year old (remote) autobiographical memories (Bonnici et al., 2012, J. Neurosci. 32:16982–16991). We found that remote memories were particularly well represented in ventromedial prefrontal cortex (vmPFC) compared to recent memories. Moreover, while both types of memory were represented within anterior and posterior hippocampus, remote memories were more easily distinguished in the posterior portion. These findings suggested that a change of some kind had occurred between two weeks and ten years in terms of where autobiographical memories were represented in the brain. In order to examine this further, here participants from the original study returned two years later and recalled the memories again. We found that there was no difference in the detectability of memory representations within vmPFC for the now 2 year old and 12 year old memories, and this was also the case for the posterior hippocampus. Direct comparison of the two week old memories (original study) with themselves two years later (present study) confirmed that their representation within vmPFC had become more evident. Overall, this within-subjects longitudinal fMRI study extends our understanding of autobiographical memory representations by allowing us to narrow the window within which their consolidation is likely to occur. We conclude that after a memory is initially encoded, its representation within vmPFC has stablised by, at most, two years later.

## Highlights

• We studied patterns of fMRI activity associated with autobiographical memories.
• We focussed in particular on vmPFC and posterior hippocampus.
• These brain regions preferentially supported remote autobiographical memories.
• Our longitudinal study showed this effect was apparent two years after encoding.
• A memory was supported by different neural populations in the hippocampus as it aged.

## 1 Introduction

Once formed, autobiographical memories of personal past episodes can stay with us for a lifetime, being available to vividly recall and re-experience anew. Whether, and how, these memory representations change over time, phenomenologically and in terms of the neural substrates that mediate them, remains an open and important question. Accounts vary about the nature of this systems-level consolidation, but there are some points of agreement. For instance, it is generally accepted that the neocortex comes to play a greater role in supporting autobiographical memories (Marr, 1971, Teyler and DiScenna, 1985, Squire, 1992, Nadel and Moscovitch, 1997, Winocur and Moscovitch, 2011, Nadel et al., 2012, Moscovitch et al., 2016). There is also agreement that autobiographical memories depend upon the hippocampus during initial encoding (Scoville and Milner, 1957). But there is also divergence, especially concerning the hippocampus. The long-standing traditional model of consolidation argues that declarative (including autobiographical) memories become less dependent on the hippocampus, eventually eschewing the need for its involvement during retrieval (Marr, 1971, Teyler and DiScenna, 1985, Squire, 1992). Alternative theories (Multiple Trace Theory, Transformation Theory, Scene Construction Theory) propose instead that the hippocampus is necessary for retrieving vivid autobiographical memories in perpetuity (Nadel and Moscovitch, 1997, Winocur and Moscovitch, 2011, Moscovitch et al., 2016, Hassabis and Maguire, 2007, Hassabis and Maguire, 2009, Maguire and Mullally, 2013, Zeidman and Maguire, 2016).

It has been difficult to adjudicate between these different views of systems-level consolidation on the basis of neuropsychological data alone, because there is evidence in favour of each theoretical position (reviewed in Winocur and Moscovitch, 2011). However, the combination of fMRI scanning of autobiographical memory retrieval in healthy volunteers and analysis methods such as multivoxel pattern analysis (MVPA; reviewed in Chadwick et al., 2012) offers potential leverage on the nature and neural evolution of autobiographical memories. Using MVPA, several studies have shown it is possible to ‘decode’ individual autobiographical memory representations from patterns of fMRI BOLD activity across voxels (Bonnici et al., 2012, Bonnici et al., 2013, Nielson et al., 2015, Rissman et al., 2016).

In a previous study, we took this further by comparing neural representations of recent (~two weeks old) and remote (~10 years old) autobiographical memories using support vector machine MVPA (Bonnici et al., 2012). There were three main findings. First, recent and remote autobiographical memory representations were distinguishable in the hippocampus, in line with theories that purport a role for the hippocampus in the vivid recall of such memories for a lifetime (Nadel and Moscovitch, 1997, Winocur and Moscovitch, 2011, Moscovitch et al., 2016, Hassabis and Maguire, 2007, Hassabis and Maguire, 2009, Maguire and Mullally, 2013, Zeidman and Maguire, 2016). Second, while autobiographical memory representations, both recent and remote, were detectable in a range of neocortical areas, the ventromedial prefrontal cortex (vmPFC) alone differentiated the two memory types, with remote memories being significantly more distinguishable there. The vmPFC has been highlighted as potentially influential for memory consolidation (Bontempi et al., 1999, Frankland and Bontempi, 2005, Nieuwenhuis and Takashima, 2011), and our finding supports this view. Third, while recent and remote autobiographical memories were represented within anterior and posterior hippocampus, remote autobiographical memories were more distinguishable in the posterior portion. Thus, like vmPFC, the hippocampus too respected the distinction between recent and remote autobiographical memories, and we further found that there was no overlap in the neural populations (as inferred from the fMRI voxel patterns) supporting both memory types. Of note, the above findings emerged in the context of a wide range of phenomenological features of the recent and remote autobiographical memories being matched (including vividness, level of detail, emotional valence), and so differences in these features cannot easily explain the differential results.

**Fig. 1:**
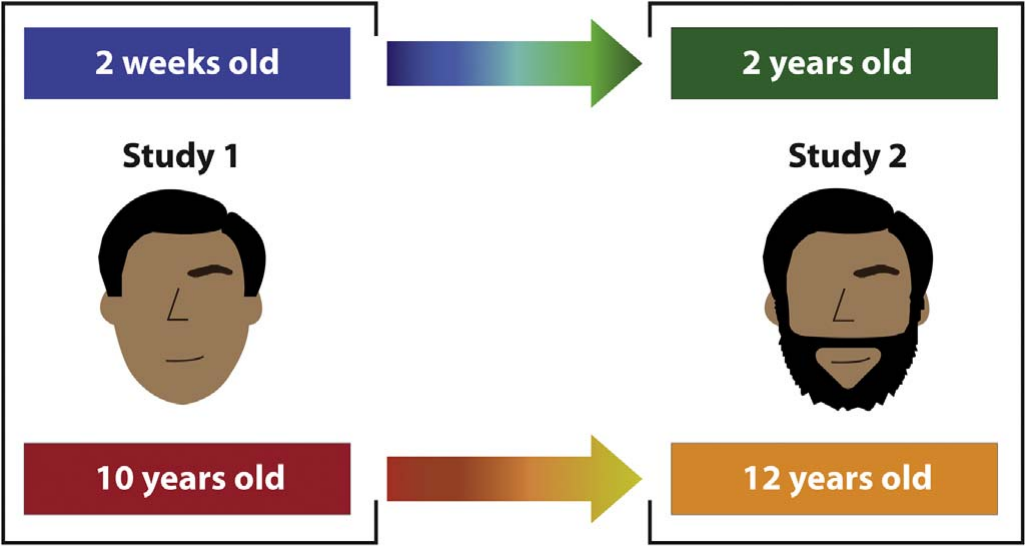
The longitudinal experimental design. In study one, participants recalled memories that were 2 weeks old and 10 years old. When they returned for study two, the 2 week old memories were now 2 years old, and the 10 year old memories were now 12 years old.

The results of Bonnici et al. (2012) strongly suggest that some change in the neural representation of autobiographical memories takes place between 2/3 weeks after formation and 10 years later. However, this time window is very large. If we are to truly elucidate the mechanisms of systems-level consolidation, then we need to narrow this window. Moreover, the recent and remote memories in Bonnici et al. (2012) were not the same memories. What is required is a longitudinal fMRI-MVPA study of autobiographical memory representations that examines the same memories over the time scale of years. While memory-related longitudinal fMRI studies have been reported (e.g., Takashima et al., 2006, Takashima et al., 2009; Hirshhorn et al., 2012), to the best of our knowledge, there is no longitudinal fMRI study of autobiographical memory. Consequently, we invited the participants from the Bonnici et al. (2012) study to return. This follow-up study was identical to that of Bonnici et al. (2012) in that it involved the same people, memories, tasks, procedures, MRI scanner, data acquisition and analysis methods. The only thing that was different was that the people and their memories were now two years older. By comparing the neural representations of the same memories at ~2 weeks old and when they were ~2 years old (and 10 and 12 years old) (see Fig. 1), we hoped to be able to put clearer time boundaries on systems-level consolidation of autobiographical memories. Given the main findings of Bonnici et al. (2012), our main interest was in examining the vmPFC and the posterior hippocampus.

## 2 Materials and methods

### 2.1 Participants

Ten of the twelve participants who took part in the original study (Bonnici et al., 2012) returned for this follow-up experiment. They were healthy, right-handed and university-educated (7 female), with a mean age of 29.1 years (SD 3.348, range 24–36 years). All had normal or corrected-to-normal vision and gave informed written consent to participation in accordance with the local research ethics committee.

### 2.2 Memories

Participants took part in the current study approximately 2 years after the first study (mean 22.3 months, SD=1.418). The decision to conduct the second phase of the experiment at this time was pragmatic – the 3T MRI scanner that was used in the first study was being replaced and so we had to conduct the follow-up experiment before the scanner was removed.

One week prior to scanning, participants were asked to recall the events they had recollected in the first study (Bonnici et al., 2012). These memories had previously been retrieved from two time periods (2 weeks or 10 years prior to scanning; three memories from each time point), therefore the memories were now either 2 years old or 12 years old (see Fig. 1). The experimenter started by giving a participant the cue that had been used for a memory in the previous scanning study and asked them to recall the relevant episode. If the cue did not prompt recall, the experimenter then provided an additional piece of information from the memory. Participants usually remembered the episode from the cue alone, and there were only a small number of occasions where a further prompt was required. The experimenter compared the description of an episode to that produced two years previously to ensure that the correct memory was being recalled and that the same details were remembered – this was true in all cases. In fact, the memory descriptions were virtually identical for studies one and two. As in study one (Bonnici et al., 2012), having described a memory, participants then rated each memory along a range of parameters, details of which are provided in the Results section.

The nature of our study design (which required sufficient training examples for the classifiers) meant that memories were recalled numerous times during study one. These memories were then recalled again in a similar manner during the current study two. Thus the memories in the follow-up study were recalled more times than those in study one. In order to verify that any difference in results that we might observe between the two studies was not due to this difference in the amount of repeated recall, we had subjects recall and describe an additional three episodes that occurred 2 years ago. These episodes happened around the same time as the now 2 year old memories of interest, but they were recalled fewer times (i.e. only during study two). If the amount of repeated recall was not an influencing factor, then the results for the now 2 year old memories that featured in study one and the ‘fresh’ two year old memories should be similar. These ‘control’ memories were elicited in exactly the same manner as study one (Bonnici et al., 2012), with an interview technique that was a standard method used in numerous previous studies (e.g., Maguire et al., 2001; Addis et al., 2004a, Addis et al., 2004b; Summerfield et al., 2009). Participants were told that the memories should unfold in an event-like way, and be very clear and vivid such that when recollecting the memory they felt as if they were re-experiencing the event. Participants were also instructed that they should provide memories that they had rarely thought about since the time the original event had occurred. General probes were given by the interviewer when required (e.g., ‘what else can you tell me about this event’). Notes were taken about each memory by the interviewer. Having described a memory, participants then rated each memory along a range of parameters as detailed in the Results section.

### 2.3 Procedure

The procedure was identical to the first study (Bonnici et al., 2012). One week after the pre-scan interview, participants returned for the fMRI scan. Prior to scanning, they were first asked how often they had thought about the memories since the session a week earlier. They were then trained to recall each memory within a 12 s recall period after viewing a word cue. There were six training trials per memory. Participants were encouraged to recall a memory as vividly as possible and to maintain the quality and consistency of this recall for the duration of the 12 s trial, and on each subsequent recall trial for this memory.

There were 9 memories – 3 memories from the first study which were now 2 years old, 3 memories from the first study which were now 12 years old, and 3 control memories that were 2 years old. During scanning participants recalled each memory fourteen times (14 trials for each of 9 memories=126 trials) split into two sessions of 63 trials and presented in a pseudo-random order, while ensuring that the same memory was not repeated twice or more in a row. On each trial, a word cue was presented for 3 s which indicated which of the nine memories a participant was required to recall. Following this, an instruction appeared on the screen indicating that participants should close their eyes and vividly recall the cued memory. Participants were instructed not to begin the recall process until this instruction appeared, and were trained on this procedure in the pre-scan session. After 12 s, an auditory tone sounded (1.5 s) signalling they should open their eyes. The participant was then required to provide ratings about the preceding recall trial using a five-key button-box. First, they rated how vivid the memory was in the preceding recall trial (on a scale of 1–5, where 1 was not vivid at all, and 5 was very vivid). Second, they rated how consistently they had recalled it relative to the original event (where 1 was not consistent at all, and 5 was very consistent). Up to 3 s was allowed for each rating, before a 4 s rest period and then the next trial. The ratings were used to select only the most vivid (ratings of 4 or 5) and most consistently recalled (ratings of 4 or 5) memories for inclusion in the MVPA analyses, ensuring that we captured genuine re-experiencing.

When trials that were not sufficiently vivid or consistent were excluded, this resulted in on average 11.07 (SD 2.61) trials for each of the three 2 year old memories, on average 11.47 (SD 2.06) for each of the three 12 year old memories, and on average 11.16 (SD 2.35) for each of the three control memories, with a mean of 97 (31 two year old, 33 twelve year old and 33 control) trials in total per participant that were entered into the MVPA analysis. There was no significant difference in trial number between the conditions (F(2,18)=0.53; p>0.05), nor did the trial number differ for the memories of interest between this and the first study for the 10 subjects that participated in both (all F(1,9) </=2.5; all p>0.05). After scanning, participants rated on a five point scale the effort required to recall the memories.

### 2.4 Functional MRI scanning

Scanning parameters were the same as those in the previous study (Bonnici et al., 2012). To reiterate, high-resolution fMRI scans were acquired in a limited volume. This included our two key regions of interest (ROIs), the vmPFC and hippocampus. The volume also included other ROIs known to be involved in autobiographical memory retrieval (Bonnici et al., 2012, Svoboda et al., 2006), entorhinal/perirhinal cortex (EPC) and posterior parahippocampal cortex (PHC), as well as the retrosplenial cortex (RSC), the temporal pole (TP) and lateral temporal cortex (LTC). A 3T Magnetom Allegra head only MRI scanner (Siemens Healthcare, Erlangen, Germany) operated with the standard transmit-receive head coil was used to acquire the functional data with a T2*-weighted single-shot echo-planar imaging (EPI) sequence (in-plane resolution=1.5×1.5 mm; matrix=128×128; field of view=192×192 mm; 35 slices acquired in interleaved order; slice thickness=1.5 mm with no gap between slices; echo time TE=30 ms; asymmetric echo shifted forward by 26 phase-encoding (PE) lines; echo spacing=560 µs; repetition time TR=3.5 s; flip angle α=90°). All data were acquired at 0° angle in the anterior-posterior axis. An isotropic voxel size of 1.5×1.5×1.5 mm was chosen for an optimal trade-off between BOLD sensitivity and spatial resolution. Further, the isotropic voxel dimension reduced re-sampling artefacts when applying motion correction. To ensure optimal data quality, images were reconstructed online and underwent online quality assurance (Weiskopf et al., 2007). For distortion correction (Hutton et al., 2002), field maps were acquired with the standard manufacturer's double echo gradient echo field map sequence (TE=10.0 and 12.46 ms, TR 1020 ms; matrix size, 64×64), using 64 slices covering the whole head (voxel size 3×3×3 mm). In addition to the functional scans, a whole brain T1-weighted 3D FLASH sequence was acquired with a resolution of 1×1×1 mm.

### 2.5 Structural MRI scanning and delineating regions of interest

Detailed structural MRI scanning was performed during study one (Bonnici et al., 2012), and those scans were utilised again in study two. As the subjects were healthy young adults we did not anticipate any gross changes in brain structure between the two studies, and comparison of whole brain FLASH scans taken during both studies confirmed this was the case. We also verified that the anatomical masks delineated on the basis of the high-resolution structural MRI scans from study one were appropriate for use with the fMRI data of study two. The only exception was the fMRI vmPFC coverage of one subject in study two which was not complete. Therefore, data reported here for the vmPFC is for n=9 subjects, and n=10 for all other brain areas. Full details of the structural scan sequences and the methods for delineating regions of interest are provided in Bonnici et al. (2012). To summarise briefly, high-resolution T2-weighted structural images (resolution=0.52×0.52×0.5 mm) were acquired on a 3T whole body MRI scanner (Magnetom TIM Trio, Siemens Healthcare, Erlangen, Germany) operated with the standard transmit body coil and 32-channel head receive coil. Images were acquired in a limited volume that included the ROIs of interest noted in the section above.

Manual segmentation of brain regions was performed using ITK-SNAP (www.itksnap.org; Yushkevich et al., 2006) on the averaged T2-weighted high-resolution structural images of each participant. Hippocampal anatomy was identified using the Duvernoy hippocampus atlas (Duvernoy, 2005). Ventromedial prefrontal cortex was delineated as one region encompassing areas where previous work demonstrated involvement in consolidation (see Nieuwenhuis and Takashima, 2011), namely BA 14, BA 25, ventral parts of areas 24 and 32, the caudal part of area 10, and the medial part of area 11. The mean number of (EPI resolution 1.5 mm^3^) voxels in each area was: HC 928.35 (SD 78.83), EPC 1464.70 (SD 189.92), PHC 581.35 (SD 79.741), RSC 543.25 (SD 125.52), TP 2647.55 (SD 569.25), LTC 3754.55 (SD 783.58), and vmPFC 1168.61 (SD 422.05). As before, segmentation of the hippocampus into its anterior and posterior portions was based on the protocol of Hackert et al. (2002), where the anterior 35% of the hippocampus was labelled as anterior and the remainder as posterior. The end of the uncus was used to delineate the border between the anterior and posterior hippocampus.

### 2.6 Image pre-processing

Image pre-processing was performed using SPM8 (http://www.fil.ion.ucl.ac.uk/spm) in an identical fashion to that of study one (Bonnici et al., 2012). The first six EPI volumes were discarded to allow for T1 equilibration effects (Frackowiak, 2004). The remaining EPI images were then realigned to correct for motion effects, and minimally smoothed with a 3 mm FWHM Gaussian kernel. A linear detrend was run on the images to remove any noise due to scanner drift (LaConte et al., 2005) using customised matlab code. Next the data were convolved with the canonical hemodynamic response function (HRF) to increase the signal-to-noise ratio (Frackowiak, 2004). This HRF convolution effectively doubled the natural BOLD signal delay, giving a total delay of approximately 12 s. To compensate for this delay, all onset times were shifted forward in time by three volumes, yielding the best approximation to the 12 s delay given a TR of 3.5 s and rounding to the nearest volume. Analysis focused on the 12 s periods of vivid recall giving a total of four functional volumes per trial.

### 2.7 MVPA

#### 2.7.1 Overview

A support vector machine (SVM) classifier was created for each region of interest. Each classifier was trained on a portion of the fMRI data relating to the three 2 year old memories and then tested on an independent set of instances of these memories. This was also the procedure for 12 year old memories, and for the control memories. This resulted in three accuracy results for each brain region, one for the 2 year old memories, one for the 12 year old memories and one for the control memories.

#### 2.7.2 Procedure

For analysis of this study's data we used a standard MVPA procedure that has been described in detail elsewhere (e.g. Chadwick et al., 2010, Chadwick et al., 2012) and that was identical to that used in the previous study (Bonnici et al., 2012). To reprise briefly, the overall classification procedure involved splitting the fMRI data into two segments: a “training” set used to train a classifier with fixed regularization hyperparameter C=1, in order to identify response patterns related to the memories being discriminated, and a “test” set used to independently test the classification performance (Duda et al., 2001), using a ten-fold cross-validation procedure. Prior to multivariate classification, feature selection (Guyon and Elisseeff, 2003) was performed on the data from the training set (thereby ensuring that this step was fully independent from the final classification, which is critical for avoiding “double-dipping”, Kriegeskorte et al., 2009). This was conducted using a standard multivariate searchlight strategy within an ROI. For a given voxel, we first defined a small sphere with a radius of three voxels centred on the given voxel (Kriegeskorte et al., 2006; see also Hassabis et al., 2009; Chadwick et al., 2010, Chadwick et al., 2012; Bonnici et al., 2012). Note that the spheres were restricted so that only voxels falling within the given region of interest were included. Therefore, the shape of the sphere and the number of voxels within it varied depending on the proximity to the region of interest's borders. This procedure then allowed the selection of the searchlight voxel set that contained the greatest degree of decoding information within the training dataset. The mean (and SD) number of voxels selected for each region (collapsed across hemisphere) was: 2 year old memories – HC: 391.96 (144.81); EPC: 758.42 (252.28); PHC: 286.92 (33.77); RSC: 266.73 (59.34); TP: 1247.72 (459.74); LTC: 1909.59 (658.37); vmPFC: 486.37 (236.02); 12 year old memories – HC: 445.11 (180.06); EPC: 820.98 (180.06); PHC: 289.25 (73.85); RSC: 273.49 (82.90); TP: 1236.17 (342.70); LTC: 1674.49 (585.49); vmPFC: 457.84 (317.82); Control memories – HC: 496.05 (101.19); EPC: 641.95 (173.48); PHC: 298.67 (99.60); RSC: 263.00 (58.29); TP: 1106.75 (483.12); LTC: 1705.19 (394.10); vmPFC: 497.47 (302.6). Using this voxel subset, the SVM classifier was trained to discriminate between, for example, the three two year old memories using the “training” image dataset, and tested on the independent “test” dataset. The classification was performed using the LIBSVM implementation (Chang and Lin, 2011). For direct comparison of studies one and two, we did not train a classifier on one study's data and test it on the other study's data. We wanted to avoid making any assumptions that precisely the same pattern of voxels would be apparent at both time points for a particular memory. As such, we ascertained the accuracy values of the most informative patterns of voxels associated with each memory/memory type at each time point within our ROIs and then compared these directly.

Standard SVMs are binary classifiers that operate on two-class discrimination problems, whereas our data involved a three-class problem (e.g. three 2 year old memories or three 12 year old memories). The SVM can, however, be arbitrarily extended to work in cases where there are more than two classes. Typically this is done by reducing the single multiclass problem into multiple binary classification problems that can be solved separately and then recombined to provide the final class prediction (Allwein et al., 2000). We used the well-established Error Correcting Output Codes approach (Dietterich and Bakiri, 1994) and computing of the Hamming distance (Hamming, 1950) as described in detail elsewhere (Hassabis et al., 2009, Chadwick et al., 2010, Chadwick et al., 2011).

#### 2.7.3 Information maps

The feature selection procedure implemented here as part of the analysis pipeline selected subsets of voxels that were most likely to carry information about the autobiographical memories. This means that for each fold of the cross-validation, a different subset of voxels was selected. As in Bonnici et al. (2012), in order to visualise the voxels selected during feature selection, an “information map” was created by simply finding all voxel sets which produced above-chance accuracy on that particular cross-validation fold in a particular ROI. These voxel sets were added together to form a single binary mask. To measure the overlap between 2 year old and 12 year old memory information maps for each participant (or the information maps between studies), we employed the widely-used Dice metric (Dice, 1945). This is a spatial overlap index where the value ranges from 0, indicating no spatial overlap between two binary mask results, to 1, indicating complete overlap. To test any overlap against chance, we randomly shuffled the positions, for example, of the 2 year old and 12 year old maps within an ROI 1000 times, and every time measured the overlap. This provided us with a null distribution of the Dice metric for that region for each participant. We could then test the actual overlap directly against this null distribution using a *t*-test.

### 2.8 Data analysis

The data analysis had three components. We first verified that the results reported in study one for the original n=12 subjects (Bonnici et al., 2012) also pertained for the n=10 subjects who returned for study two. Second, we analysed the data for study two. Third, we then directly compared the data from studies one and two. Comparisons of classifier accuracy values for the different memory conditions, and for comparing memories with themselves across time, were conducted using repeated measures ANOVAs and significant results were subsequently interrogated using two-tailed paired *t*-tests. As in Bonnici et al. (2012) classifier results for the left and the right hemispheres were highly similar, and therefore the data we report here are collapsed across hemispheres. The classifier accuracy values for each brain region were compared to chance (which was 33% in this experiment). Given that we were only interested in whether results were significantly above chance, one tailed *t*-tests were used. Given the results of study one (Bonnici et al., 2012), the main focus of our analysis was the hippocampus (including its anterior and posterior portions) and the vmPFC. Where relevant, and for completeness, we also report data from the other ROIs. A statistical threshold of p<0.05 was employed throughout.

## 3 Results

### 3.1 Behavioural data

Having recalled the memories in the interview session one week before scanning, participants rated them along a range of parameters which included vividness, level of detail, emotional valence and effort required for memory retrieval. The mean ratings are shown on Table 1. Analysis of the study one data for the 10 participants confirmed that, as with the original study (n=12; Bonnici et al., 2012), there were no significant differences between the ratings for the then 2 week old and 10 year old memories (all t(9). </=1.5; p>0.05). When the memories were recalled again in the current study two, again, there were no significant differences between the ratings for the now 2 year old and 12 year old memories (all t(9)</=1.16; p>0.05). Moreover, the ratings for the 2 year old memories (all t(9)</=0.7; p>0.05) and the 12 year memories (all t(9)</=−0.83; p>0.05) did not differ significantly from those for the control 2 year old memories.

**Table 1:** Memory characteristics.

**Measure**	**Study 1**		**Study 2**			**Study 1 vs Study 2**			
	**2 Week**	**10 Year**	**2 Year**	**12 Year**	**Control Memories**	**2 Week vs** **2 Year**		**10 Year vs** **12 Year**	
	**mean (SD)**	**mean (SD)**	**mean (SD)**	**mean (SD)**	**mean (SD)**	**t (9)**	**p**	**t (9)**	**p**
Recall frequency between interview and scan	1.1 (0.23)	1.03 (0.1)	1 (0)	1 (0)	1 (0)	1.4	0.19	1	0.34
Vividness	4.56 (0.38)	4.5 (0.39)	4.52 (0.33)	4.5 (0.37)	4.61 (0.3)	0.36	0.73	0.05	0.96
Level of detail	4.43 (0.45)	4.20 (0.61)	4.30 (0.4)	4.13 (0.59)	4.2 (0.48)	0.72	0.49	0.25	0.81
1st/3rd person perspective	1 (0)	1.06 (0.14)	1 (0)	1 (0)	1 (0)	0	1	1.5	0.17
Emotional valence	3.19 (0.32)	3.17 (0.17)	3 (0.16)	3 (0.22)	3 (0)	2.25	0.051*	1.86	0.096
Active/static event	1 (0)	1.03 (0.1)	1 (0)	1 (0)	1 (0)	0	1	1	0.34
Effort of recall (post-scan rating)	1.27 (0.34)	1.53 (0.57)	1.67 (0.63)	1.53 (0.59)	1.7 (0.64)	−3.75	0.005*	0.01	0.99

N=10 participants. Ratings were on a scale of 1–5, where 1 was the minimum and 5 the maximum. For emotional valence: 1,2=negative, 3= neutral, 4,5= positive. For 1st/3rd person perspective: 1=1st person, 2=3rd person. For active/static event: 1=active, 2=static.

* =significantly different.

We then directly compared the data from the two studies, and statistical comparisons are shown on Table 1. The same autobiographical memories when 2 weeks old (study one) and 2 years old (study two) did not differ significantly on ratings of vividness, level of detail and they were all in a first person perspective and comprised active events. The memories were regarded as marginally more positive in study one, although the difference was only 0.19 out of ratings of 1–5. The memories in study one were rated as being easier to recall, although again, the difference was very small, being just 0.4 out of ratings of 1–5. There were no significant differences across any of the ratings for the other set of memories when they were 10 years old (study one) and 12 years old (study two).

### 3.2 MVPA – summary of study one (now n=10)

**Fig. 2:**
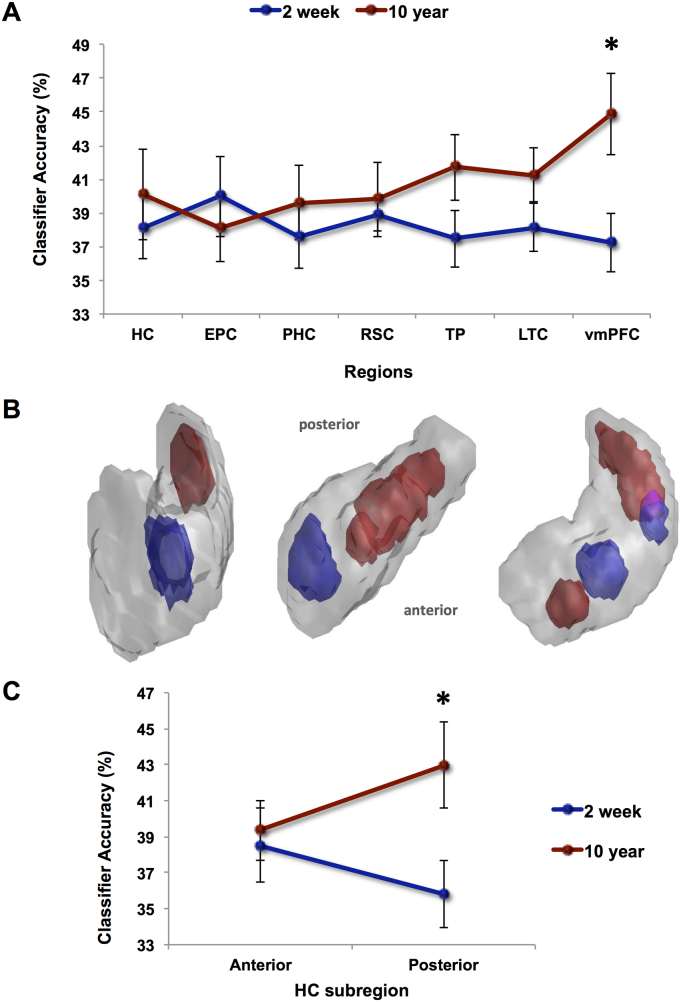
MVPA results for recent and remote autobiographical memories in study one. **A**. Hippocampus (HC), entorhinal and perirhinal cortices (EPC), parahippocampal cortex (PHC), retrosplenial cortex (RSC), temporal pole (TP), lateral temporal cortex (LTC), and ventromedial prefrontal cortex (vmPFC) were examined. Chance=33%. Recent (blue line) and remote (red line) autobiographical memories were represented in medial temporal regions, including the hippocampus, while in vmPFC remote memories were particularly distinguishable; *p<0.05. The difference between recent and remote memories in TP just failed to reach significance. Error bars represent ±1 standard error of the mean. **B.** Information maps in the hippocampus for recent (shown in blue) and remote (shown in red) autobiographical memories comprised the voxel sets that produced above-chance classification accuracy. The information maps for three example participants are shown superimposed upon 3D images of their right hippocampus. Areas in pink denote where the information maps for recent and remote memories overlapped. **C.** MVPA results for anterior and posterior subregions of the hippocampus (HC). Above-chance (chance=33%) classification was apparent in anterior and posterior hippocampus for recent (blue line) and remote (red line) memories. Nevertheless, classification accuracies were significantly higher in the posterior hippocampus for remote memories; *p<0.05. Error bars represent ±1 standard error of the mean. (For interpretation of the references to color in this figure legend, the reader is referred to the web version of this article.).

The results of study one for the 10 subjects who returned for the current experiment did not differ in any significant way from the original study (Bonnici et al., 2012). For convenience, the key findings are summarised in Fig. 2 and presented below.

#### 3.2.1 Two week old memories

We explored whether it was possible to predict which of the three 2 week old memories was being recalled solely from the pattern of activity across voxels. For each brain region of interest a classifier was first trained on a portion of the fMRI data relating to the three 2 week memories and then tested on an independent set of trials of these memories (see Materials and methods). If it was possible to discriminate between the three memories from the patterns of fMRI activity, then the classifier would produce a classification result significantly above chance (33%). Classifiers operating on voxels in all seven ROIs were able to distinguish between the three 2 week old autobiographical memories significantly above chance, all p<0.05 (Fig. 2A, blue line): HC: t(9)=2.727; EPC: t(9)=2.932; PHC: t(9)=2.461; RSC: t(9)=6.289; TP: t(9)=2.649; LTC: t(9)=3.556; and vmPFC: t(8)=2.421.

#### 3.2.2 Ten year old memories

Having established that above-chance decoding of 2 week old autobiographical memories was possible in our regions of interest, we next considered the three 10 year old memories. As with the 2 week old memories, for each brain region of interest a classifier was trained on a portion of the fMRI data relating to the three 10 year old memories and then tested on an independent set of trials of these memories. Classifiers operating on voxels in the seven ROIs were able to distinguish between the three 10 year old autobiographical memories significantly above chance, all p<0.05 (Fig. 2A, red line): HC: t(9)=2.651; EPC: t(9)=2.526; PHC: t(9)=3.004; RSC: t(9)=3.077; TP: t(9)=4.411; LTC: t(9)=5.105; and vmPFC: t(8)=4.890. Our results, therefore, showed that the remote 10 year old memories were represented not only in cortical areas, but also in the medial temporal lobe, including the hippocampus.

#### 3.2.3 Direct comparisons of 2 week old and 10 year old memories

As in Bonnici et al. (2012), the classification accuracies for 2 week old and 10 year old memories did not differ significantly in the hippocampus (t(9)=−0.535, p>0.05), while there was a significant difference in vmPFC, with higher classification accuracy values for the 10 year old memories (t(8)=−2.934; p<0.05). This confirms the pattern that is apparent in Fig. 2A where 2 week old and 10 year old autobiographical memories were distinguishable from patterns of hippocampal (and medial temporal) activity. Memories were also distinguishable in cortical areas, but the 10 year old remote memories were more discriminable in vmPFC.

#### 3.2.4 Spatial distribution of information in vmPFC and hippocampus

Having established that the 2 week old and 10 year old autobiographical memories were represented in our key regions of interest, vmPFC and hippocampus, we then proceeded to explore the spatial distribution of the discriminating voxels within each of these regions. Specifically, we wanted to determine whether the voxel patterns that supported the 2 week old memories overlapped with those supporting the 10 year old memories. Information maps for 2 week old and 10 year old memories were created from the voxel sets that produced above-chance classification accuracy (see Materials and methods). To measure the overlap between recent and remote memory information maps we used the Dice metric.

We first examined the vmPFC and found that the Dice metric for overlap between the 2 week old and 10 year old memory information maps was 0.25. To determine whether this overlap was significantly different from what would be expected by chance, we randomly shuffled the positions of the 2 week and 10 year maps within vmPFC 1000 times, and every time measured the overlap. This provided a null distribution of the Dice metric for vmPFC. When the actual score was tested against this null distribution, it was not significantly different from chance (t=−0.043, p>0.05), suggesting that the voxel patterns (and by inference the underlying neuronal populations) that supported the 2 week old memories overlapped with those supporting the 10 year old memories in vmPFC.

By contrast, the Dice metric for the 2 week old and 10 year old memory information maps in the hippocampus, where the null distribution was also determined using the permutation testing procedure, was lower (0.18) than for vmPFC. When this score was tested against the null distribution of the Dice metric that had been calculated for the hippocampus, it was significantly lower than would be expected by chance (t=−3.201, p<0.05), suggesting that the information maps for 2 week old and 10 year old autobiographical memories in the hippocampus did not overlap very much. Visual inspection of the information maps of the participants (see examples in Fig. 2B) suggested a separation down the long axis of the hippocampus for 2 week old and 10 year old autobiographical memories. To interrogate this further, the hippocampus was subdivided into anterior and posterior portions (see Materials and methods), and MVPA analyses were repeated in these portions separately (Fig. 2C). As in Bonnici et al. (2012) there was no difference in classification accuracy in anterior hippocampus for 2 week and 10 year old memories (t(9)=−0.326, p>0.05), with both types of memory represented there. Classification accuracies were significantly higher in the posterior hippocampus for 10 year old memories compared to 2 week old memories (t(9)=−2.436; p<0.05).

In summary, analysis of the study one Bonnici et al. (2012) data from the ten participants who returned for study two mirrored the original (n=12) data precisely. Recent 2 week old memories and remote 10 year old memories were represented in the hippocampus. There was a difference in cortical areas, with 10 year old memories being better represented in vmPFC compared to the 2 week old memories. Within the hippocampus there was also a distinction, with anterior hippocampus representing 2 week old and 10 year old memories, while the 10 year old autobiographical memories were better represented in posterior hippocampus compared to the recent 2 week old memories. The next question we addressed was what happened to these specific memories when participants returned two years later.

### 3.3 MVPA – study two

#### 3.3.1 Two year old memories

**Fig. 3:**
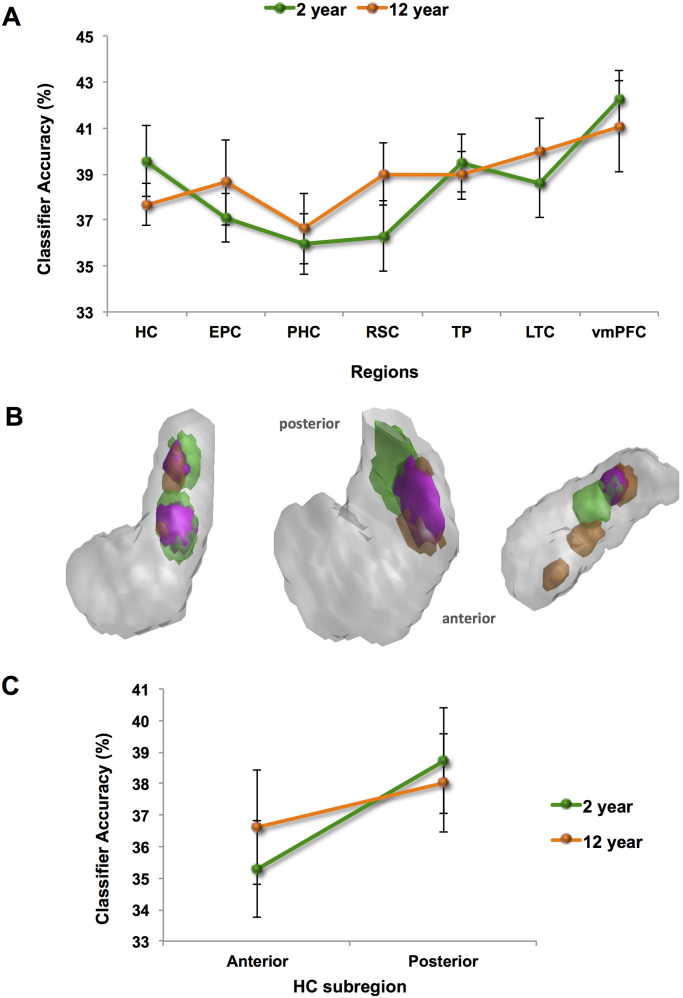
MVPA results for recent and remote autobiographical memories in study two. **A**. Hippocampus (HC), entorhinal and perirhinal cortices (EPC), parahippocampal cortex (PHC), retrosplenial cortex (RSC), temporal pole (TP), lateral temporal cortex (LTC), and ventromedial prefrontal cortex (vmPFC) were examined. Chance=33%. Two year old (green line) and 12 year old (orange line) autobiographical memories were represented in medial temporal regions, including the hippocampus. In study two, this was also the case for vmPFC. Error bars represent ±1 standard error of the mean. **B.** Information maps in the hippocampus for 2 year old (shown in green) and 12 year old (shown in orange) autobiographical memories comprised the voxel sets that produced above-chance classification accuracy. The information maps for three example participants are shown superimposed upon 3D images of their right hippocampus. Areas in pink denote where the information maps for the 2 year old and 12 year old memories overlapped. **C.** MVPA results for anterior and posterior subregions of the hippocampus (HC). No differences between memory types were apparent in either the anterior or posterior hippocampus. Error bars represent ±1 standard error of the mean. (For interpretation of the references to color in this figure legend, the reader is referred to the web version of this article.).

We first explored whether it was possible to predict which of the three 2 year old memories was being recalled solely from the pattern of activity across voxels. For each brain region of interest a classifier was first trained on a portion of the fMRI data relating to the three 2 year old memories and then tested on an independent set of trials of these memories (see Materials and methods). Classifiers operating on voxels in all seven ROIs were able to distinguish between the three 2 year old autobiographical memories significantly above chance, all p<0.05 (Fig. 3A, green line): HC: t(9)=4.282; EPC: t(9)=3.913; PHC: t(9)=2.238; RSC: t(9)=2.171; TP: t(9)=5.117; LTC: t(9)=3.774; and vmPFC: t(8)=7.242.

#### 3.3.2 Twelve year old memories

Having established that above-chance decoding of 2 year old autobiographical memories was possible in our regions of interest, we next considered the three 12 year old memories. As with the 2 year old memories, for each brain region of interest a classifier was trained on a portion of the fMRI data relating to the three 12 year old memories and then tested on an independent set of trials of these memories. Classifiers operating on voxels in the seven ROIs were able to distinguish between the three 12 year old autobiographical memories significantly above chance, all p<0.05 (Fig. 3A, orange line): HC: t(9)=5.185; EPC: t(9)=3.040; PHC: t(9)=2.368; RSC: t(9)=4.357; TP: t(9)=5.671; LTC: t(9)=4.732; and vmPFC: t(8)=4.081.

Our results, therefore, showed that even when autobiographical memories were formed 2 years or 12 years prior to scanning, they were represented not only in cortical areas, but also in the medial temporal lobe, including the hippocampus.

#### 3.3.3 Direct comparisons of 2 year old and 12 year old memories

Comparing classification accuracies for 2 year old and 12 year old autobiographical memories showed no significant differences in medial temporal lobe (MTL) structures [HC, EPC, PHC; F(1,9) 2.608, p>0.05; paired *t*-tests (all p>0.05): HC: t(9)=0.984; EPC: t(9)=−0.649; and PHC: t(9)=−0.316]. However, unlike study one, when cortical areas were considered (RSC, TP, LTC, vmPFC), no significant effect was present [F(1,8)=0.709, p>0.05); paired *t*-tests (all p>0.05): RSC: t(8)=−1.375; TP: t(8)=0.409; LTC: t(8)=−0.947; and vmPFC: t(8)=0.639]. This confirms the pattern in Fig. 3A, where the previous difference in vmPFC classifier accuracies that was evident between the memory types two years ago (Fig. 2A) is now no longer evident.

In Bonnici et al. (2012), we also performed an additional confirmatory analysis in order to ascertain if decoding of memories was possible in a way that generalised across the three memories within each memory set. We found that the hippocampal (t(11)=5.255, p<0.001) and vmPFC (t(11)=5.274, p<0.001) classifiers could distinguish between the recent 2 week old memories and the remote 10 year old memories significantly above chance. Interestingly, when we performed the same analysis on the study two data, this was no longer the case, presumably because all of the memories were now ‘remote’ (HC: t(9)=0.98, p=0.35; vmPFC: t(8):=0.46. p=0.66).

#### 3.3.4 Spatial distribution of information in vmPFC and hippocampus

We next examined the spatial distribution of information within vmPFC and hippocampus using the Dice metric for the 2 year old and 12 year old memory information maps and calculations of the null distributions using the permutation testing procedure outlined previously.

Considering vmPFC, we found that the Dice metric for overlap between the 2 year old and 12 year old memory information maps was 0.24, which was not significantly different than would be expected by chance (t(8)=−1.097, p>0.05). This suggests that the voxel patterns (and by inference the underlying neuronal populations) that supported the 2 year old memories overlapped with those supporting the 12 year old memories in vmPFC. This is not surprising given that similar overlap was found in study one when the memories were 2 weeks old and 10 years old. The next question concerned the hippocampus, where previously a difference had been found. Now, however, two years later, the Dice metric for overlap of the 2 year old and 12 year old memories was 0.24. In contrast to the results of the previous study, this was not significantly lower than would be expected by chance (t(9)=0.069, p>0.05). This suggests that by the time of study two, the voxel patterns that responded to the 2 year old memories overlapped with those supporting the 12 year old memories in the hippocampus. Visual inspection of the information maps appears to confirm this (see examples in Fig. 3B). Interrogating this further by examining the anterior and posterior hippocampus separately (Fig. 3C), as in study one there was no difference in classification accuracy in anterior hippocampus for 2 year old and 12 year old memories (t(9)=0.896, p>0.05), with both types of memory represented there. However, unlike before, classification accuracies were now not different in the posterior hippocampus for 2 year old compared to 12 year old memories (t(9)=−1.718; p>0.05).

#### 3.3.5 Control analyses

We conducted two types of control analyses. First, as in study one (Bonnici et al., 2012), we examined accuracy values in control (i.e. not memory-related) cortical regions in the left and right lateral posterior visual cortex. Classifier accuracies for 2 year old and 12 year old memories were at chance, i.e. it was impossible to predict which memories were being recalled from the patterns of activity across voxels there (collapsed across left and right posterior visual cortex; 2 year old: t(9)=0.8, p>0.05; 12 year: t(9)=0.945, p>0.05). This shows that our classification analysis was not biased towards invariably producing above-chance accuracies.

Second, we compared the classification accuracies for the 2 year old and 12 year old memories with the control memories, which were also two years old but had been recalled fewer times (i.e. only in study two). There were no significant differences in vmPFC (F(2,16)=0.54; p>0.05), hippocampus (F(2,18)=0.66; p>0.05), or indeed any of the other ROIs (all F(2,18)</=2.39; p>0.05). This shows that differences in recall frequency did not have any appreciable effect on classifier accuracy.

In summary, even after two years had passed since study one, it was still possible to detect representations of individual autobiographical memories, be they 2 years or 12 years old, in patterns of fMRI activity across voxels in a range of ROIs, including the hippocampus. However, three notable changes were evident in study two. First, there was no difference in classifier accuracies for the vmPFC for the now 2 year old and 12 year old memories. Second, there was greater overlap in the information maps for the 2 year old and 12 year old memories within the hippocampus. Third, classifier accuracy values in the posterior hippocampus were now not significantly different between the two memory types. Thus, although the same memories were recalled in studies one and two, some changes seemed to have occurred in how they were represented in the brain. We next assessed whether these apparent differences were borne out when the data from the two studies were compared directly.

### 3.4 MVPA – comparison of studies one and two

Results from the two studies suggest that representations of the remote memories that were 10 years old and then 12 years old remained largely unchanged during the intervening years and so direct comparison of the data should not reveal any significant differences between the two time points for these memories. By contrast, disparities between the two studies seem to revolve around the more recent memories that were 2 weeks old and then 2 years old, with the results from study two suggesting that their representations had become more like the remote 10 year old/12 year old memories. A such, direct comparison of the same memories when they were 2 weeks old and 2 years old should show a difference in classifier accuracy values for the vmPFC, with the memories more readily detected there when 2 years old. In the hippocampus, there should be little overlap in the information maps for the memories at the two time points. Related to this lack of overlap, the memories at two years old might also be more readily detected in the posterior hippocampus.

**Fig. 4:**
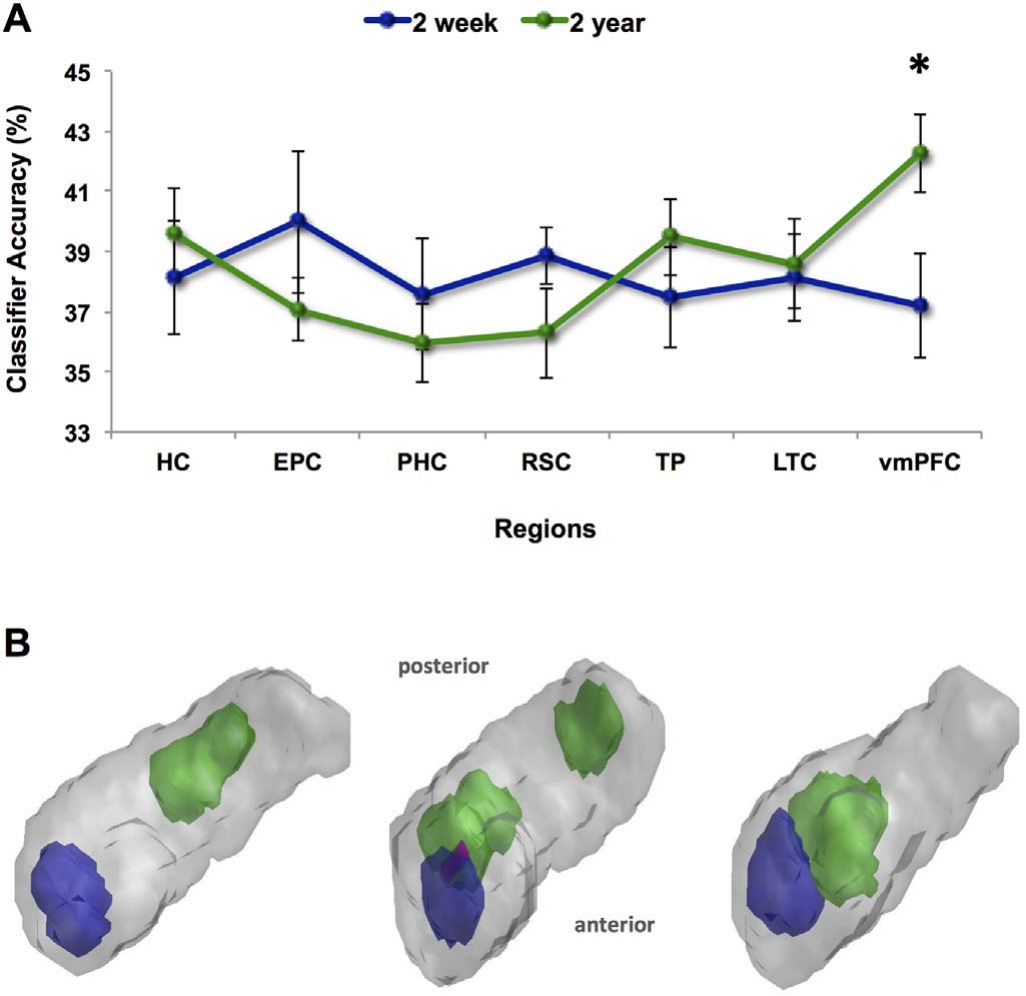
MVPA results for the comparison of the same memories when 2 weeks old and 2 years old. **A.** Hippocampus (HC), entorhinal and perirhinal cortices (EPC), parahippocampal cortex (PHC), retrosplenial cortex (RSC), temporal pole (TP), lateral temporal cortex (LTC), and ventromedial prefrontal cortex (vmPFC) were examined. Chance=33%. The memories when two weeks old (blue line) and 2 years old (green line) were represented in medial temporal regions, including the hippocampus, while memories were more easily distinguishable when they were 2 years old in vmPFC; *p<0.05. Error bars represent ±1 standard error of the mean. **B.** Information maps in the hippocampus for the memories when 2 weeks old (shown in blue) and 2 years old (shown in green) comprised the voxel sets that produced above-chance classification accuracy. The information maps for three example participants are shown superimposed upon 3D images of their right hippocampus. Areas in pink denote where the information maps for the memories at the two different time point overlapped. (For interpretation of the references to color in this figure legend, the reader is referred to the web version of this article.).

**Fig. 5:**
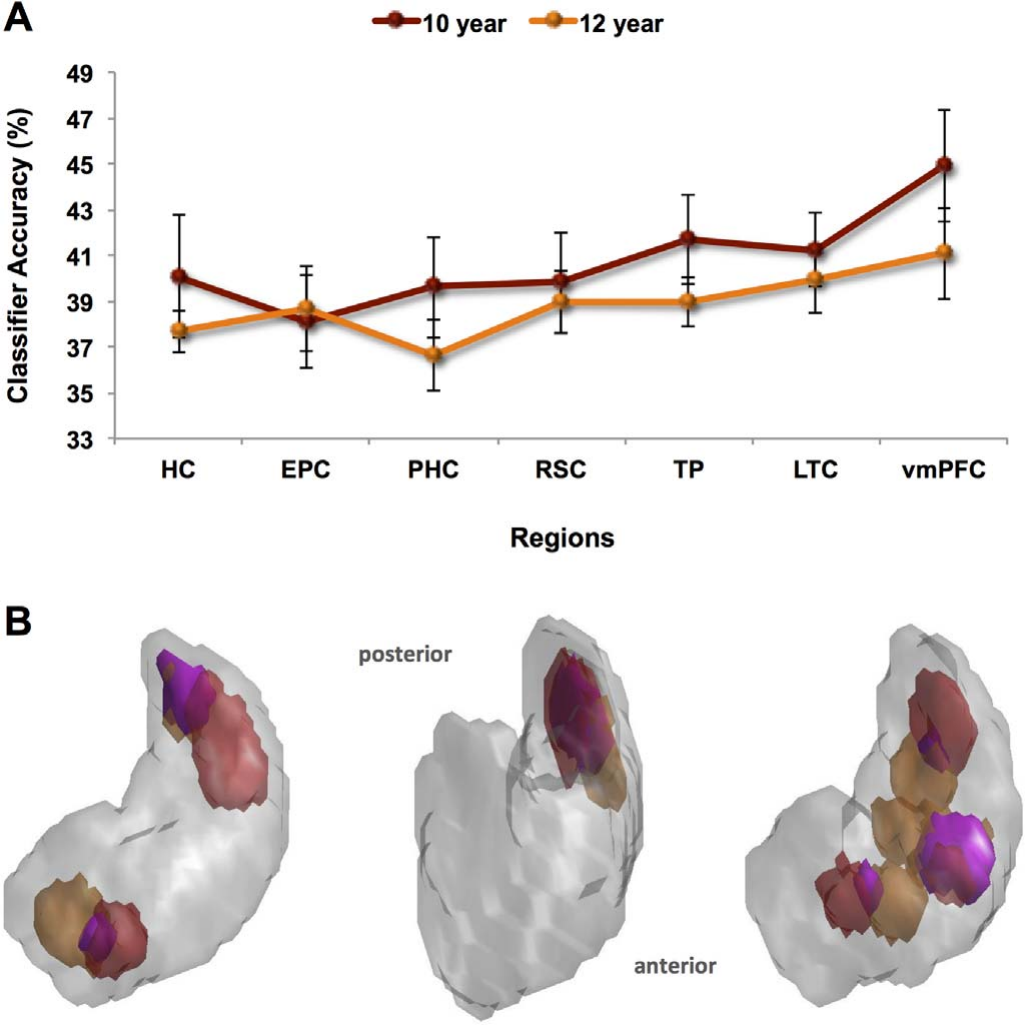
MVPA results for the comparison of the same memories when 10 years old and 12 years old. **A.** Hippocampus (HC), entorhinal and perirhinal cortices (EPC), parahippocampal cortex (PHC), retrosplenial cortex (RSC), temporal pole (TP), lateral temporal cortex (LTC), and ventromedial prefrontal cortex (vmPFC) were examined. Chance=33%. When memories were ten years old (red line) and then 12 years old (orange line), they were represented in medial temporal regions, including the hippocampus, and this was also the case for the vmPFC. Error bars represent ±1 standard error of the mean. **B.** Information maps in the hippocampus for the memories when 10 years old (shown in red) and 12 years old (shown in orange) comprised the voxel sets that produced above-chance classification accuracy. The information maps for three example participants are shown superimposed upon 3D images of their right hippocampus. Areas in pink denote where the information maps for the memories at the two different time point overlapped. (For interpretation of the references to color in this figure legend, the reader is referred to the web version of this article.).

Considering first the vmPFC, we observed a significant interaction between study and memory type (F(1,8)=5.533; p<0.05). This was due to vmPFC classifier accuracies being significantly greater for 10 year old compared to 2 week old memories in study one (t(8)=2.394; p=<0.05) and, crucially, greater for 2 year old compared to 2 week old memories (t(8)=−2.263, p<0.05). This latter vmPFC difference, between exactly the same memories when they were examined two years apart, is evident in Fig. 4A. When the remote memories were compared at 10 years old and at 12 years old, no differences were apparent, as shown on Fig. 5A (t(8)=1.191, p>0.05).

Turning next to the hippocampus, the Dice score for the information map overlap for the memories when they were 2 weeks and 2 years old was 0.15. This was significantly lower than would be expected by chance (t(9)=−2.415, p<0.05), indicating that the information maps in the hippocampus did not overlap very much. Visual inspection of the information maps confirmed this (see examples in Fig. 4B). By contrast, the information maps for the remote memories when they were 10 years old and 12 years old were not significantly lower than would be expected by chance (t(9)=−1.672, p>0.05) and indeed visual inspection of the information maps indicated a good deal of overlap (see examples in Fig. 5B). Finally, as expected, there was no interaction between study and memory type for anterior hippocampal classifier accuracy values (F(1,9)=0.035, p=0.855). For posterior hippocampus, the interaction between study and memory type just failed to reach significance (F(1,9)=4.024, p=0.07).

## 4 Discussion

We twice examined neural representations of specific autobiographical memories with a two year gap between visits, in order to leverage a better understanding of the time-scale involved in system-level consolidation. This longitudinal approach yielded three main findings which revolved around the memory representations that were 2 weeks old in study one and 2 years old in the second study. First, these memories were more easily distinguished in vmPFC when examined two years later. Second, in the hippocampus, there was little overlap in the information maps for these memories when they were 2 weeks and 2 years old. Third, related to this lack of overlap, there was no difference in the detectability of the memory representations in the posterior hippocampus at 2 years old compared to the even more remote 12 year old memories. Thus, over time, some changes seemed to have occurred in how the memories were represented in the brain. Consequently, this narrows considerably the estimated time window for autobiographical memory consolidation, suggesting it may be largely complete by, at most, two years after memory formation.

Other than the two year passage of time, the two studies were highly similar – involving the same people, memories, tasks, procedures, MRI scanner, data acquisition and analysis methods (Bonnici et al., 2012). Therefore, general protocol differences cannot explain the changes in the 2 week old-2 year old memories, and notably representations of the more remote memories that were 10 years old in study one and 12 years old in study two, did not change, suggesting that change was not an automatic by-product of our longitudinal paradigm. Another concern might be that participants were in fact recalling the interview one week before scanning. However, if this were the case, then it would have been true for all memories, even the very remote 10/12 years old memories, and this would have precluded finding any differential effects, which we in fact observed in study one and between studies one and two. The same argument holds for the suggestion that our results reflect ‘re-encoding’ – again, if this were the case, it would have applied to all memories and presumably prevented differential findings. One difference between the two studies was the extent of repeated recall of the memories during scanning. Obviously, the nature of our study design (which required sufficient training examples for the MVPA analysis) meant that memories in the follow-up study were recalled more times than those in study one. Perhaps this led to the differential between-study findings, as repetition is known to affect neural representations (e.g., Xue et al., 2010; but see Svoboba and Levine, 2009). If repetition per se was a key factor, then we would have expected to see some differential effects for the 10/12 year old memories also, which we did not. Moreover, in order to examine this issue directly, in the second study we included new 2 year old memories that were recalled at the same frequency as the memories in study one. Despite a difference in the recall frequency between these new 2 year old memories and the 2 year old memories that were 2 weeks old in study one, the same findings pertained, which strongly suggests that our findings are not explicable by the extent to which the memories were rehearsed. It may be that vividly re-experienced autobiographical memories are not subject to the same repetition effects that have been associated with simpler stimuli (Xue et al., 2010).

An obvious place to look for differences over time in autobiographical memory recall is in the subjective phenomenological features of the memories (Addis et al., 2004a, Gilboa et al., 2004). In the first study we specifically included neutral/positive memories that participants were able to vividly recall, and this was the case for the then 2 week old and 10 year old memories. Despite all the phenomenological features being matched between the recent and remote memories (see Table 1 here and also Bonnici et al., 2012), differences in their neural representation were observed. When studied two years later, again the subjective ratings for the now 2 year old memories and the 12 year old memories did not differ from each other, or indeed from the control 2 year old memories. Direct comparison of the memories across studies showed that the same autobiographical memories when 2 weeks old and 2 years old did not differ significantly on ratings of vividness, level of detail and they were all in a first person perspective and comprised active events. The memories were regarded as marginally more positive in study one, although the difference was only 0.19 out of ratings of 1–5. The memories in study one were also rated as being easier to recall, although again, the difference was very small, being just 0.4 out of ratings of 1–5. While we cannot rule out the possibility that these minor disparities influenced, or even caused, the differential MVPA findings for these memories, we find it hard to believe that such small margins of difference could have large neural effects. However, we cannot know for certain. Moreover, we measured explicit subjective ratings in our studies, and it may be that differences between time points on an implicit level that could not be expressed by participants, were in some way associated with the neural changes (Winocur and Moscovitch, 2011, Moscovitch et al., 2016).

An alternative explanation for the Bonnici et al. (2012) findings was offered by Berkers and van Kesteren (2013). They suggested that participants might have inserted new episodic details particularly into remote memories during the prescan interview and that this could have influenced hippocampal engagement. Study two allowed us to address this possibility. When the memory descriptions were examined in terms of objective details, there were no differences between study one and study two, in fact, the memory descriptions were virtually identical, making it unlikely that this factor caused the neural changes that we observed.

Another way to consider the effects of consolidation is to examine the functions of the brain regions involved and try to deduce from that what variables might be pertinent. In our studies, the vmPFC was the key region that showed changes over time. Both between and within autobiographical memories, remote memories were more easily distinguished there, with ‘remote’ meaning memories that were more than two years old. However, there is still no consensus about the functions of the vmPFC. It is has been linked with a dizzying array of domains (reviewed in Delgado et al., 2016; Nieuwenhuis and Takashima, 2011; Szczepanski and Knight, 2014; Gilboa et al., 2006) including, but not limited to, inhibitory control, decision making, emotional and social control, value, motivation and reward-based processing, pre-retrieval guidance of memory searches, integrating retrieved information together, post-retrieval monitoring and the formation and implementation of schema. On the basis of our data, we cannot ‘solve’ the vmPFC. All we can do is note that our findings could be associated with the increased need to coordinate the retrieval and integration of elements of remote autobiographical memories from other neocortical areas, which are then funnelled into the hippocampus to be reconstructed. These processes may also require schema to guide them, and possibly to monitor the results for appropriateness (to avoid confabulation – Gilboa et al., 2006). Indeed, increased functional and effective connectivity between the hippocampus and prefrontal cortex has been reported during the reconstruction and elaboration of autobiographical memories (McCormick et al., 2015). Whether the vmPFC is involved in all of these actions, or whether subregions within vmPFC undertake specific tasks, is as yet unknown. Nevertheless, our findings here suggest a time frame for representational change that could be explored further to help inform debates about the contributions of the vmPFC.

The other brain area to show time-related changes in autobiographical memory representations was the hippocampus. As in study one (Bonnici et al., 2012), individual autobiographical memories were decodable from hippocampal activity irrespective of their remoteness. More specifically, we found that the anterior hippocampus contained decodable information about all of the memories. This accords with recent views showing that the anterior hippocampus in particular seems to be associated with scene-based cognition, including the reconstruction in perpetuity of the vivid and detailed scenes that comprise autobiographical memories (Zeidman and Maguire, 2016; see also Maguire and Mullally, 2013). By contrast, as with the vmPFC, remote memories were more easily distinguished in the posterior hippocampus, with a significant disparity evident in study one between the 2 week old and 10 year old memories which had disappeared two years later. Functional differences down the long axis of the hippocampus are now well-documented, although there is as yet no agreement about the precise nature of this functional divide (Fanselow and Dong, 2010; Poppenk et al., 2013; Strange et al., 2014; Zeidman and Maguire, 2016). It may be that the effects we noted in posterior hippocampus reflect the retrieval or processing of spatial contexts that, for remoter memories, may require more re-instantiation than those for very recent autobiographical memories. Of note, the interaction between study and memory type just failed to reach significance for the posterior hippocampus. This may indicate that whatever consolidation process the memories underwent was perhaps not quite completed at the two year time point when the memories were re-examined. Alternatively, it could be that with additional participants, this effect may have been more robust.

One hippocampal result that was significant and particularly intriguing was our observation that the neural populations within the hippocampus (as indexed by voxel information maps) that supported an autobiographical memory differed depending on the memory's age. For instance (Fig. 4B), a memory when 2 weeks old was supported by a separable neural population when 2 years old. This seems to run contrary to ideas about reactivation and neurons that ‘fire together wire together’ (Hebb, 1949, Oudiette et al., 2013, Tambini et al., 2010). Although Multiple Trace Theory asserts that a different ‘trace’ is laid down in the hippocampus every time an autobiographical memory is recalled (Nadel and Moscovitch, 1997; see also Gilboa et al., 2004), it is often assumed that the same neural population that supported a memory initially becomes active again, in whole or in part, at retrieval (e.g. pattern completion). Our data suggests this may not be the case, and in its strongest interpretation could indicate that perhaps there are no indexes/pointers (e.g., Marr, 1971) to or for specific memories stored in the hippocampus. Consequently, what happens at retrieval might be coordinated from elsewhere (the vmPFC?) and the machinery of the hippocampus works on whatever information it receives without regard to the previous neuronal populations that processed a memory in the past.

In this longitudinal study we assessed autobiographical memories at different intervals and were able to set an outer limit for systems-level consolidation of, at most, two years. What remains unknown is the time course of consolidation before 2 years. This transition could be linear, step-wise or have some other form, and may well be completed long before two years. Future longitudinal studies, while challenging to conduct, will be needed to address this important point. More work is also required to examine not only the very vivid and easily retrievable memories on which we focussed here, but also those memories that are more difficult to retrieve and are less vivid, as this may shed further light on the roles of the vmPFC and posterior hippocampus. Finally, we did not address here the important issue of connectivity between brain regions that support autobiographical memories (Söderlund et al., 2012), and this will also be essential to consider in order to achieve a full understanding of systems-level consolidation.

## Competing financial interests

The authors declare no competing financial interests.
